# Enhancing Genetic Gain through Genomic Selection: From Livestock to Plants

**DOI:** 10.1016/j.xplc.2019.100005

**Published:** 2019-10-16

**Authors:** Yunbi Xu, Xiaogang Liu, Junjie Fu, Hongwu Wang, Jiankang Wang, Changling Huang, Boddupalli M. Prasanna, Michael S. Olsen, Guoying Wang, Aimin Zhang

**Affiliations:** 1 Institute of Crop Science/CIMMYT-China, Chinese Academy of Agricultural Sciences, Beijing 100081, China; 2 CIMMYT-China Tropical Maize Research Center, Foshan University, Foshan 528231, China; 3 CIMMYT-China Specialty Maize Research Center, Shanghai Academy of Agricultural Sciences, Shanghai 201400, China; 4 Institute of Genetics and Developmental Biology, Chinese Academy of Sciences, Beijing 100101, China; 5 CIMMYT (International Maize and Wheat Improvement Center), ICRAF Campus, United Nations Avenue, Nairobi, Kenya

**Keywords:** genomic selection, genetic gain, open-source breeding, genomic prediction, molecular marker, livestock breeding

## Abstract

Although long-term genetic gain has been achieved through increasing use of modern breeding methods and technologies, the rate of genetic gain needs to be accelerated to meet humanity's demand for agricultural products. In this regard, genomic selection (GS) has been considered most promising for genetic improvement of the complex traits controlled by many genes each with minor effects. Livestock scientists pioneered GS application largely due to livestock's significantly higher individual values and the greater reduction in generation interval that can be achieved in GS. Large-scale application of GS in plants can be achieved by refining field management to improve heritability estimation and prediction accuracy and developing optimum GS models with the consideration of genotype-by-environment interaction and non-additive effects, along with significant cost reduction. Moreover, it would be more effective to integrate GS with other breeding tools and platforms for accelerating the breeding process and thereby further enhancing genetic gain. In addition, establishing an open-source breeding network and developing transdisciplinary approaches would be essential in enhancing breeding efficiency for small- and medium-sized enterprises and agricultural research systems in developing countries. New strategies centered on GS for enhancing genetic gain need to be developed.

## Teaser

Accelerating genetic gain is needed to meet humanity's demand for plant products. Genomic selection, which has been largely used in livestock, can be used to enhance genetic gain in plants. The genomic selection strategies reviewed in this paper for plants include field and environment management, optimum model development, cost reduction, platform and network construction, open-source breeding, and transdisciplinary approaches.

## Introduction

To meet the demand for plant-based products, plant breeding has been systematically evolving from art to science with the advent and development of genetics and genomics (Xu, 2010). Taking three major United States crops—maize, wheat, and soybean—as examples, the evolution has contributed to positive linear increases in average yield during the period 1930–2012 (USDA-National Agricultural Statistics Service, 2013). However, crop yield growth (genetic gain) has been slowing down. For example, annual maize yield growth has reduced from 2.20% for the period 1960–1990 to 1.74% for 1990–2010, and is expected to be further reduced to 1.33% for the period 2010–2050 (Pardey et al., 2014). Taking all major crops together, the annual yield growth rates are insufficient to produce the 70% more crop products that are required by 2050 to meet the increasingly growing demand (Tester and Langridge, 2010, Fischer et al., 2014). Therefore, enhancing genetic gain is crucial to filling the gap between demand and production.

**Figure 1:**
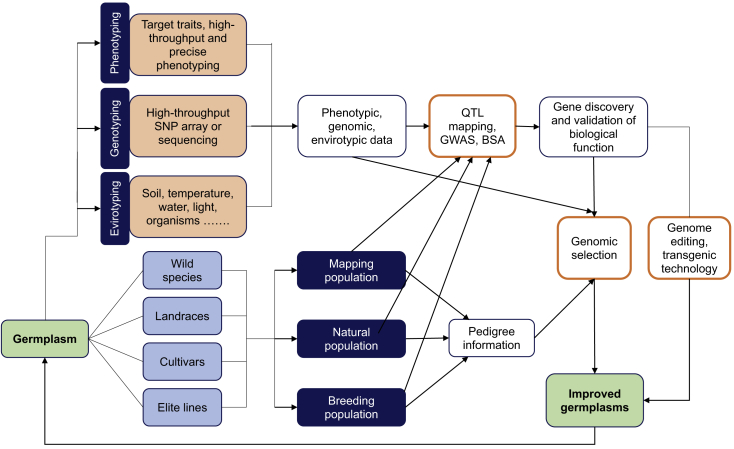
Flowchart of Background and Knowledge Relevant to Genomic Selection. GWAS, genome-wide association study; QTL, quantitative trait loci; BSA, bulked sample analysis (Zou et al., 2016).

In early breeding stages, breeders intentionally selected plants and animals, mainly based on phenotypes that may include a few key yield-related traits, to achieve the genetic gain for target traits. As the development of quantitative genetics and statistics continued, best linear unbiased prediction (BLUP) was proposed (Henderson, 1985, Henderson, 1990, Searle et al., 1992) and used to estimate breeding values for evaluating and selecting better potential species in animal breeding by using the phenotypic and pedigree information of the offspring or other relatives of the sire. However, this selection process was time-consuming and cost-intensive because evaluating offspring's phenotypes is often too expensive, and phenotyping can be only done when the offspring of potential breeding sires grow up. Later the BLUP method was gradually applied in plant breeding, which, however, has not obtained the popularity similar to that in animal breeding (Bernardo, 1996, Durel et al., 1998, Dutkowski et al., 2002, Xiang and Li, 2003, Viana et al., 2010). Using molecular markers associated with target traits, marker-assisted selection (MAS) was proposed and used for selection of the traits controlled by genes with relatively large effects. Using genetic markers to select the candidate sires has been successfully integrated into livestock breeding programs as a time-saving and highly efficient breeding strategy (Dekkers and Hospital, 2002, Goddard and Hayes, 2009). However, many complex traits such as yield are controlled by many genes or quantitative trait loci (QTL) each with relatively small effects, interacting with environments. Their individual effects are too small to be efficiently captured (Bernardo and Yu, 2007, Nakaya and Isobe, 2012), although their combining effects could be statistically significant. As an alternative approach for breeding complex traits and minor genetic effects, genomic selection (GS) was proposed, with the hypothesis that with high-density markers each trait-related locus should be associated with at least one marker for the purpose of choosing top-ranked lines based on individuals' genomic estimated breeding values (GEBVs) (Meuwissen et al., 2001, Heffner et al., 2009, Jonas and de Koning, 2013). In addition to efficiently capturing both major and minor gene effects using whole-genome markers, GS has at least two other advantages in comparison with traditional MAS, including no need to unearth the QTL related to target traits and no need of phenotyping during later breeding stages (Nakaya and Isobe, 2012). Moreover, GS is an embodiment of a whole-genome strategy with the development of traditional forward genetics, including mapping and functional validation of candidate genes (Figure 1). GS has been considered as one of the seven key post-1990 “bandwagons” that take a critical place in the course of plant improvement, along with transgenic cultivars, QTL mapping, association mapping, phenomics, envirotyping, and genome editing (Bernardo, 2016).

**Figure 2:**
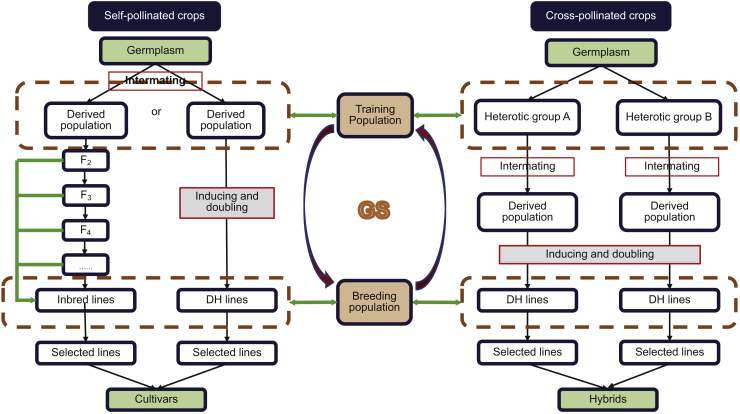
Genomic Selection Procedure in Breeding Programs for Self-Pollinated (Left) and Cross-Pollinated (Right) Crops. A training or reference population is used to estimate marker effects and then the genomic estimated breeding values of each individual in breeding populations, and the selected candidate lines can be regarded as founders for the next cycle of breeding. GS, genomic selection; DH, doubled haploid.

In GS, genome-wide markers are used to estimate their effects through optimum statistical models, then GEBVs are calculated for each individual to select potential elite lines. More precisely, two types of populations are required in GS, a training population (TP) (also called reference population) that is composed of a cohort of individuals with both genotypic and phenotypic data, and a breeding (or testing) population (BP) that consists of candidate breeding lines with genotypic data only. Data from TP are used to train a statistical model to estimate the effect of each assayed marker and then calculate the estimated breeding values for each genotyped individual in BP to rank the lines without phenotyping. Furthermore, these reserved individuals can be served as parental lines that may intermate with each other to pyramid favorable alleles for the next cycle of selection (Jonas and de Koning, 2013, Desta and Ortiz, 2014; Figure 2). Although selection in breeding can be processed based on GEBVs, breeders sell varieties by their commercial value, which may not be justified only by their GEBVs.

Improvement, or response to selection, can be evaluated by the genetic gain achieved with the relevant selection methods including GS. Genetic gain can be defined by the quantity of increase in performance that is obtained through selection programs, and its expected value per year can be measured as: Δ*G* = *i* σ_*A*_ *r*_MG_/*t*, where Δ*G* is the expected genetic gain, *i* is intensity of selection, σ_*A*_ is genetic SD, namely the square root of additive genetic variance, *r*_MG_ is selection accuracy (measured by the correlation between breeding values and GEBVs), and *t* is breeding cycle time. In the context of phenotypic selection, however, the *r*_MG_ is equivalent to the square root of the narrow sense heritability (*h*), and thus Δ*G* = *i* σ_*A*_ *h*/*t* (Meuwissen, 2003, Heffner et al., 2010, Bassi et al., 2016, Xu et al., 2017). To enhance genetic gain, several approaches related to its formula components can be considered in breeding programs, including increasing intensity of selection, enlarging genetic SD, improving prediction/selection accuracy or heritability, and shortening breeding cycle time (Table 1; Xu et al., 2017). Besides, the relationship of traits related to the target environment or user and the costs of breeding program have been proposed as additional factors affecting genetic gain (Hickey et al., 2017). The relationship can be included as a part of components that affect heritability while the cost will determine selection intensity, as high cost will reduce the population size with which breeders can work.

**Table 1:** Factors Affecting Genetic Gain and Potential Contributors Associated with Genomic Selection in Plants.

Components	Subcomponents	Contributors
Population	Population type	Bi- and multiparental populations, natural populations, mating-design populations, multiple-hybrid populations, population structure and relationship
	Population size	*In vitro* culture, DH technology, costs in phenotyping, genotyping, and envirotyping
	Novel germplasm introduction	Exotic germplasm, transgene, genome editing, mutation, gene introgression
	Selection proportion	Breeding project, population size, heritability, TP/BP ratios
	Selection method	Phenotypic selection, GS, MARS, integrated selection
	Selection index	Indices based on breeding objectives, trait priorities
	Germplasm used to estimate GEBVs	Population type, size, relationship, and structure
Genotype	Molecular marker	Marker type, marker density, LD between QTL and marker, functional versus neutral markers, genome distribution
	Targeted genes	Transgene, mutation, genome editing
Heritability	Field management	Experimental design, field management, trial site selection, uniform agronomic practice, environment management and control, precision phenotyping, envirotyping
	Estimation	Population types, mating design, traits: major genes controlled or minor genes controlled
GS model	Statistical model	Genetic effects, genotype-by-environment interaction, rrBLUP, Bayesian models, machine learning, pedigree information, non-additive effect, fixed effect, multivariate model, among others
Breeding scheme	Breeding program	Breeding objective, selection criteria, selection scheme, target-environmental selection, breeding cost
	Integrated breeding platform	GS, MAS, MARS, genome editing, DH, seed DNA-based genotyping
	Off-season screening	Speed breeding, greenhouse, winter nursery

DH, doubled haploid; MAS, marker-assisted selection; MARS, marker-assisted recurrent selection; rrBLUP, ridge-regression best linear unbiased prediction; GS, genomic selection; LD, linkage disequilibrium; QTL, quantitative trait locus; TP, training population; BP, breeding population.

## Genomic Selection in Plants: Bottlenecks and Constraints

GS has been verified with great potential to improve genetic gain in plant and animal breeding, especially in livestock breeding (Farah et al., 2016, Kariuki et al., 2017, Mehrban et al., 2017, Weller et al., 2017). When GS was introduced as a conceptual and theoretical method, the potential of GS was simulated in dairy cattle (Schaeffer, 2006). Largely due to higher value of each individual, greater reduction in generation interval and, thus, higher genetic gain, GS has been widely used in livestock breeding, starting with dairy cattle in 2008 and on a very large scale in pigs, sheep, beef cattle, and chickens later on (Wolc et al., 2015, Lu et al., 2016, Wiggans et al., 2017, Georges et al., 2019). GS in dairy cattle has reduced the generation interval from 7 years, which is required for the bulls to have sufficient daughters with milk records to estimate breeding values accurately, to 12 months when the bulls could be selected for artificial insemination based on their GEBVs. The early selection with large TPs has doubled genetic gain over the past decade, compared with the selection based on progeny testing (García-Ruiz et al., 2016), and GS in US Holstein bulls has resulted in significant increases in net merits/year from US$19.01 for 2000–2004 to $47.72 for 2005–2009 and to $84.87 for 2010–2014 (Wiggans et al., 2017). In plant breeding, however, GS has been implemented largely in multinational seed companies, and the most optimal GS strategies are species-dependent and breeding program-dependent (Voss-Fels et al., 2019), although numerous studies have been reported for many major crops with support by government funds (Supplemental Table 1). To enhance genetic gain, GS-assisted breeding programs should take all affecting factors as shown in Table 1 into consideration to achieve maximum benefits and high returns. Therefore, bottlenecks and constraints in the future GS breeding programs should be fully examined.

The requirements for livestock GS breeding programs include an affordable and adequate genotyping platform, availability of extensive pedigree records and years of progeny testing, less structured populations allowing utilization of molecular markers with substantial and long-term effects, relatively simplified but functional breeding pipelines in which selection based on additive genetic effects can produce beneficial consequences, and cooperation between institutes and enterprises to exploit and perform original strategies into a subsistent breeding program. These requirements indicate what we can improve in plant GS breeding (Jonas and de Koning, 2013). Extensive and large-scale use of GS in plants needs to reduce GS costs involved in breeding programs, develop cost-effective genotyping, phenotyping, and envirotyping platforms, create diverse and updatable TPs, develop highly efficient and multifunctional genomic prediction models, shorten breeding cycle time and speed up the breeding process, build up a strong decision support system, and establish open-source breeding programs (Table 2). Beyond that, high-throughput and precision phenotyping, using purebred lines and developing predictive models, should be considered more seriously when GS is implemented in plant breeding, because genotype-by-environment interaction (GEI) has significant effects on phenotypic performance in plants but a limited effect on livestock, including dairy cattle whose breeding cohorts are raised in facilities allowing for a better condition management (Jonas and de Koning, 2013). In addition, each crop species may have many breeding programs, and breeders may also want to design GS for specific breeding objectives with many different types of populations to work with.

**Table 2:** Comparison of Genomic Selection between Livestock and Plants.

	Livestock	Plants	Shared
**Feasibility**			

Value-chain	Higher individual value with higher investment return	Lower individual value with low investment return	Reduced cost; improved efficiency and thus genetic gain
Cost	More tolerant to high cost	Less tolerant to high cost	Reduced cost and breeders' affordability for large-scale GS
Benefit	More benefit from early selection and reduced generation interval	Off-season selection; less benefit from early selection and reduced generation interval	Early selection; reduced generation interval; accumulating favorable alleles for complex traits

**Platforms**			

Genotyping	Relatively higher cost acceptable due to higher individual value; easier DNA/RNA extraction; available pedigree records and progeny testing data	Relatively lower cost required due to lower individual value; complicated DNA/RNA extraction; limited pedigree and progeny testing data	Flexible, low-cost, high-throughput markers and platforms; significant, functional markers and genes; good marker coverage; high-density markers
Phenotyping	Movable individuals; individual-based; usually smaller numbers	Fixed individuals; group- or population-based; larger numbers	High-throughput, precision, and low-cost protocols and platforms
Envirotyping	Relatively uniform sites and environments; easier to measure, control, and standardize	Diverse locations and environments; harder to measure, control, and standardize	Controlled and managed environments; modeled and optimized growth and development factors
Informatics and decision support	Less demanding as data are relatively few due to limited population types, numbers, and sizes	Highly demanding as data are sizeable due to populations of diverse types, larger numbers, and bigger sizes	Data collection, storage, and mining; modeling; making decision; big data-driven breeding

**Training and breeding populations**			

Population type	Largely heterozygous	Open- versus self-pollinated; natural versus designed; hetero- versus homozygous; temporary versus permanent; inbreeding versus distant	Desired for more training population types with known population structure
Population number	Small	Larger and multiple populations from specific parents or natural collection	Desired for more training and breeding populations
Population size	Small in pedigree and limited by siblings	Various sizes from small to large, species-dependent	Desired for large population sizes
Sharing and updating	Not sharable via seeds; not updatable via permanent or regenerated populations	Easier to share populations via seeds or tissue; updatable for permanent or regenerated populations	Sharable G-P-E information; updatable pedigrees and specific individuals

**Factors affecting genetic gain**			

Genetic variation	Not possible to discover genetic variation via homozygous processing; not manageable for fine mapping and gene cloning via linkage mapping; difficult to create new alleles via mutation	Easier to discover genetic variation via homozygous processing; manageable for fine mapping and gene cloning via linkage mapping; easier to create new alleles via mutation due to controlled inbreeding	Developing markers for full-genome coverage; unlocking hidden genetic variation from closely related species; identifying markers and genes via GWAS; creating new alleles via gene transfer and genome editing
Heritability	Relatively higher due to weaker environmental effects and smaller experimental errors; environments are easier to be controlled or managed	Relatively lower due to stronger environmental effects and larger experimental errors; environments are more difficult to be controlled or managed	Improvable via managed trials with controlled environmental effects and errors
Selection intensity	Lower potential for increasing via larger population size or lower selection rate	Greater potential for increasing via larger population size and lower selection rate	Improvable using larger population size and lower selection rate
Breeding cycle time	Not manageable for rapid homozygous process; less sensitive to photoperiods; not or less adaptable to speed breeding via off-season trials or tissue culture; extremely sensitive to early selection	Rapid homozygous process via DH; probably sensitive to photoperiods; more adaptable to speed breeding via off-season trials and tissue culture; less sensitive to early selection	Shortened or accelerated cycle by early selection, shortened generation interval and accelerated generation, via clones and modified metabolism/pathways and adjusted growth
Statistical models	Less significant GEI; defined or known population structure; one model probably fit for the same population type	Very significant GEI; diverse levels of population structure; different models needed for diverse population types	Various statistical models: BLUP, GBLUP, rrBLUP, BayesA, wBSR, RKHS, BayesB, BayesCπ; biological effects: non-additive factors, epistasis, GEI, growth and development, networks, pathways

**Breeding strategies**			

Germplasm evaluation	Not possible to maintain germplasm for a long term; non-renewable; pedigree-based evaluation	Easier to maintain for a long term under managed conditions; renewable; continuous and repeat evaluation with data accumulated	Evaluated for trait donors and gene discovery; identifying associated markers and genes via GWAS for GS; creating populations for model training and breeding
Prebreeding	Less important and less manageable	Important and practical	Desired for creating new germplasm more manageable to breeders
Stress tolerance	Abiotic: managed by controlled environments; biotic: managed by gene modification, surgery, internal medicine therapy	Abiotic: managed via environmental control, improved tolerance, chemical control or regulation; biotic: managed by integrated control and improved tolerance	Adjusted and enhanced adaptation and tolerance to abiotic and biotic stresses
Open-source breeding	More applicable for breeding parents or parental populations	Suitable for all cases in plant breeding	Sharing G-P-E information and even genetics and breeding materials; sharing GS-related platforms across livestock and plants

BLUP, best linear unbiased prediction; GBLUP, genomic BLUP; GEI, genotype-by-environment interaction; G-P-E, genotype–phenotype–environment; GS, genomic selection; GWAS, genome-wide association study; RKHS, reproducing kernel Hilbert space; rrBLUP, ridge-regression BLUP; wBSR, weighted Bayesian shrinkage regression.

There are several primary factors that affect GS significantly, including marker density, population size, statistical models, genetic relationship between TP and BP, population structure, and accuracy of phenotyping (Table 1). With empirical data or simulation, these key primary factors have been evaluated for their effects on prediction accuracy. Prediction accuracy varies from model to model because the models have different prior assumptions and diverse hypotheses on the distribution of marker effects (Heslot et al., 2012, Ogutu et al., 2012, de los Campos et al., 2013, Liu et al., 2018). TP design plays an important role in GS by contributing to a high level of prediction accuracy or improving BP diversity under precise and efficient breeding projects (Isidro et al., 2015, Zhang et al., 2017b). Generally, high marker density can ensure that one trait-related QTL is in LD with at least one marker, and consequently achieve high predictive performance (Zhao et al., 2012, Combs and Bernardo, 2013). The difference in allele frequencies between TP and BP can affect prediction accuracy, as allele frequencies can affect the estimated genomic relationship matrix when GBLUP models are implemented (VanRaden, 2008, Su et al., 2012). In addition, the accuracy and cost in phenotyping, genotyping, and envirotyping may affect heritability estimation for targeted traits and marker effect estimation, and thus prediction accuracy (Xu et al., 2017). Compared with QTL mapping and genome-wide association study (GWAS), however, GS can not only capture the minor effects of insignificant markers through optimal models but also facilitate their use in breeding programs.

Management of environments and reduction of general costs are two additional challenges to GS breeding in plants (Table 2). To breed diverse varieties for different specific environments, plant breeders need to work with large numbers of populations each with many plants, increasing the cost significantly in genotyping and other GS procedures. As each plant is usually fixed in a specific location/site for its whole life, the microenvironments around the plant will have significant impacts on its growth and development and, thus, on phenotyping. To reduce the effects of specific microenvironments on individual plants, breeders need to use the average phenotypic performance of a group of individual plants to represent a specific genotype, significantly increasing phenotyping cost. On the other hand, controlled or well-managed environments are required to minimize the disturbing effects of environments and genotype × environment interactions on phenotyping (Xu, 2016).

## Improving Prediction Accuracy of Genomic Selection

Regarding the aforementioned strategies aimed at enhancing genetic gain, one of them aims to strengthen GS per se with improved predictive accuracy under cost-benefit balance (Tables 1 and 2). In fact, the precision of estimated marker effects holds an important position in the course of GS prediction, and any approaches by which accuracy and stability of predictive marker effects can be improved have potential to augment prediction accuracy and thus enhance genetic gain. In general, prediction accuracy is influenced largely by marker density, population size and structure, TP–BP relationship, heritability, and genetic models. Therefore, prediction accuracy (*r*_GM_) can be expressed using the following formula:

*r*_GM_ ≈ a*x*_1_ + b*x*_2_ + c*x*_3_ + d*x*_4_ + e*x*_5_,

where *x*_1_ is associated with marker density, *x*_2_ population size and structure, *x*_3_ TP–BP relationship, *x*_4_ heritability, and *x*_5_ genetic models; a to e are constants associated with the five corresponding variables *x*1 to *x*5, which may not be linearly regressed with *r*_GM_. As refining field management for improved heritability estimation is more related to breeding technologies; heritability-related issues will be discussed in the next section.

### Improving Prediction Accuracy with High-Density Markers

Marker density has been shown to be an extremely important factor affecting prediction accuracy. Generally, high marker density can have benefit to augment prediction accuracy until prediction accuracy reaches a plateau and does not increase further as marker density increases (Cao et al., 2017, Lee et al., 2017, Xu et al., 2018, Juliana et al., 2019). Moreover, required marker density will vary with plant species, and population types and sizes. The marker density required for outcrossing species is higher than that for self-pollinated species (e.g., Liu et al., 2018, Juliana et al., 2019). The marker numbers required for natural populations are normally higher than those for biparental populations (Liu et al., 2018, Hao et al., 2019). The reason for this phenomenon is that natural populations usually have significant population structure with high LD between adjacent markers, and thus high-density markers should be required to make sure that each trait-associated locus can be in LD with at least one marker (Meuwissen et al., 2001, Wang et al., 2017a). Biparental populations have clear genetic structure and limited recombination incidents that can be produced in the process of population development. Therefore, a moderate marker density could be enough to ensure that at least one marker can be in linkage with gene-related locus (Smith et al., 2008, Lorenzana and Bernardo, 2009). Along with the dramatic cost reduction in genotyping, the genotyping-by-sequencing (GBS) strategy, including reduced-representation sequencing (Miller et al., 2007, Baird et al., 2008), whole-genome resequencing (Huang et al., 2009), and genotyping by target sequencing (GBTS) (Guo et al., 2019a), has become one of the most promising approaches. The term GBS can be generalized to include all multiplexing PCR or targeted sequencing methods such as used in GenoPlexs/Ampliseq (Stevanato et al., 2017, Zhang et al., 2018) and GenoBaits/SureSelect (Neves et al., 2011, Jupe et al., 2014; Rawat et al., 2016, Guo et al., 2019a). Such genotyping platforms provide a better option for GS to increase marker density with low cost. Moreover, plant breeders have implemented GBS approaches with high-density markers into empirical GS, such as selection in wheat and maize breeding populations for harnessing minor variation while equally achieving better prediction accuracy (Poland et al., 2012, Crossa et al., 2013, Rutkoski et al., 2014, Zhang et al., 2015, Gorjanc et al., 2016). With significant reduction of genotyping cost, marker number or density may no longer be our concern, so that we can use one all-purpose, high-density marker panel for all types of populations.

### Increasing Population Sizes and Balancing the TP–BP Relationship

Three key learnings from implementing GS in livestock and plants are all about TPs: large population size, close relationship with selection candidates, and frequent update (Voss-Fels et al., 2019; Table 2). Investigation of the effect of the TP/BP ratio indicated that prediction accuracy reached at a stable level when the ratio was 1-fold (Cao et al., 2017), although the optimized ratio may change with the population sizes used in modeling and need to be evaluated with large population sizes. Simulation has been widely used to generate corresponding TP and BP datasets when a cross-validation scheme is implemented for training statistical models (Habier et al., 2007, Jia and Jannink, 2012, Daetwyler et al., 2013). By establishing training samples using clustering, graphic network analysis, and genetic mating scheme, designed TP outperformed random sampling (Guo et al., 2019b). In addition, resampling can be used to generate different sets of training and testing data from a real dataset with a large population size (Cao et al., 2017, Zhang et al., 2017a, Liu et al., 2018). For quality phenotyping and an adequate accurate GEBV (0.5), 5000 and 2500 individuals are required in TP for low-heritability traits with *h*^2^ = 0.2 and *h*^2^ = 0.4, respectively (Voss-Fels et al., 2019). To maintain or optimize accuracy across selection stages, GS models should be frequently updated (Podlich et al., 2004, Heffner et al., 2011, Yabe et al., 2017), largely due to the decreased marker-QTL LD caused by increased recombination events. This section will focus on the relationship between TP and BP.

TP and BP should be close enough to share long-range haplotypes, making the GS most accurate (Cooper et al., 2014b, Lorenz and Smith, 2015, Meuwissen et al., 2016). Prediction accuracy in GS was remarkably higher when TP and BP had a closer genetic relationship (Schulz-Streeck et al., 2012, Zhang et al., 2017a). GS experiments for the grain yield with diverse panels composed of fixed maize lines illustrated that prediction accuracy is lower between groups than within groups (Windhausen et al., 2012), and more accurate prediction was achieved with closely related populations (Heslot et al., 2015). Several studies highlighted the importance of increasing the relatedness by including more related crosses in TP rather than increasing the TP size by adding unrelated or less-related crosses (Riedelsheimer et al., 2013, Jacobson et al., 2014, Lorenz and Smith, 2015, Brandariz and Bernardo, 2019). In an extensive wheat study, 2992 lines from 44 F_2:4_ bi- and triparental populations were genotyped using 25 000 segregating single-nucleotide polymorphisms (SNPs) and grown in four field locations. Prediction accuracies of yield generally increased with training-set size. Higher prediction accuracies were obtained using related crosses in training and validation sets compared with using unrelated crosses, indicating the importance of training-panel design (Edwards et al., 2019). In another wheat GS study, a moderate prediction accuracy was achieved for a highly structured population (Habyarimana, 2016), compared with a higher prediction accuracy with less structured populations (Isidro et al., 2015, Spindel et al., 2015, Liu et al., 2018). Besides, prediction accuracy could become negligible (too low) when unrelated lines were included in the TP (Crossa et al., 2014). However, when more related individuals were used to train models, more accurate prediction could be achieved (Crossa et al., 2014.; Endelman et al., 2014, Duangjit et al., 2016).

To enhance genetic gain, the population relationship should be taken seriously into account when breeders are prepared with implement GS strategy to augment the potential of selection, which may determine whether a GS project would be successful. However, continuously using closely related populations to achieve better prediction would narrow down the genetic basis, reduce genetic variation that would contribute to our future selection response, and thus slow down the genetic gain that would be achieved in long-term GS (Jannink et al., 2010, Hickey et al., 2019, Moeinizade et al., 2019). Therefore, the TP–BP relationship should be balanced and optimized by considering genetic gain for both short-term and long-term selection (Li et al., 2008). One of the strategies to achieve such a balance is to include associated genetic regions and variants targeted by molecular markers, by which diverse BPs distantly related with the TP can be used. On the other hand, using a part of a population as TP (phenotyping a small section in the target environment) to predict the rest of the population as BP in off-seasons or off-locations could be an alternative strategy that takes advantage of both the close TP–BP relationship and diverse populations. In this way, off-season or off-location trials can be used for both generation advancement and selection through large-scale GS.

### Integrating Marker Effects and GWAS Results into Prediction Models

Alongside the rapid development of molecular marker systems, various molecular breeding strategies have been proposed and applied in plant breeding by using genome-wide markers. Functional markers identified and validated in previous studies can be used as fixed effects in the model to improve prediction accuracy (Figure 1). Markers located near genes, affecting gene function, or known to be causal mutations have been used to improve the accuracy of genomic predictions. By incorporating prior biological knowledge about known genomic regions that are more likely to affect the trait of interest, prediction accuracy was improved (MacLeod et al., 2016). By adding causative variants and removing less informative markers, a 1.4 percentage-point gain across traits for Holstein cattle was achieved in the United States national genomic evaluation (Wiggans et al., 2016). By including the five markers located on chromosomes BTA8, BTA9, BTA13, BTA17, and BTA27 with dominance effects on male fertility as fixed effects in the predictive models, predictive correlations increased to 0.403 from 0.340 for that without inclusion. Multikernel models fitting all the functional SNP classes together with the five major markers exhibited predictive correlations of around 0.405 (Nani et al., 2019).

Prediction accuracy is related to the actual effects of chromosome segments that can be represented by the markers. For complex traits with low heritability, prediction accuracy is relatively low, resulting in high non-additive variance or an effect that is hardly captured by molecular markers. Therefore, the significant loci detected by GWAS (Spindel et al., 2016), functional genes verified by molecular biological experiments (Arruda et al., 2016), or assumptive major QTL based on simulation studies (Bernardo, 2014) have been considered as fixed effects in GS models to understand whether including candidate major QTL can improve the prediction accuracy for agronomic traits with low heritability. Significant QTL identified by GWAS can be used to modify statistical models in GS to improve prediction accuracy. The most significantly associated markers can be designed as fixed effects in the model along with polygenic background, and individual SNPs that have prominent and strong association signals can efficiently improve GS (Spindel et al., 2016, Bian and Holland, 2017).

Using a dataset consisting of 1500 Jersey bulls with sire conception rate (SCR) records and 95 000 SNPs, linear and Gaussian kernel-based models were used to fit both the entire SNPs and the subsets of SNPs either significantly associated with SCR or located within or close to annotated genes. The entire SNP set exhibited predictive correlations of around 0.30. SNPs marginally associated with SCR or genic SNPs both achieved higher predictive abilities than their counterparts using random SNPs (Rezende et al., 2019). Predictions for milk fatty acid traits in cows using a multipopulation reference and a traditional GBLUP model resulted in average gains in prediction reliability of 10% points in the Dutch, 8% points in the Danish, and 1% points in the Chinese populations compared with predictions based on population-specific references. By incorporating GWAS results (substantial proportions of genetic variation on *Bos taurus* chromosomes 14, 19, and 26) as genomic features, the revised GBLUP led to further increases in prediction reliability (up to 13%–38% points across different populations) (Gebreyesus et al., 2019). In fact, using a small quantity of significant markers as genotypic matrix in the models can achieve a more accurate prediction. In brief, potential inbred lines can be selected from breeding populations based on GEBVs. By integrating GS with MAS or GWAS with a few of gene-related markers, prediction accuracy and thus genetic gain can be improved. Using simulated traits from diversity panels in maize and sorghum, ridge-regression best linear unbiased prediction (rrBLUP) models that include fixed-effect covariates tagging peak GWAS signals were evaluated. The inclusion boosted prediction accuracy for only 60 out of the 216 genetic architectures simulated, and in several instances increased both the variability of prediction accuracies and the bias of GEBVs (Rice and Lipka, 2019). Therefore, the performance of such a GS model should be explored on a trait-by-trait basis prior to its implementation into a breeding program. Integrating GS with GWAS or QTL mapping can be implemented as a new strategy in crop breeding, and the accumulated sheer amount of experience can provide useful guidelines for accelerating the breeding process.

### Including GEI and Non-additive Effects in Prediction Models

Various statistical models can be implemented in GS to train genotypic and phenotypic data to determine whether the marker effects are precisely estimated (Table 2). Many reports offer new models or provide comparative model analyses (Heslot et al., 2012, de los Campos et al., 2013, Cuevas et al., 2014, Ceron-Rojas et al., 2015, LeCun et al., 2015) and the relationship of different modeling approaches (Morota and Gianola, 2014). Models have been proposed with different prior hypothesis on marker effect distribution and distinct parametric or non-parametric controls for the purpose of efficient and proper dimensionality (Crossa et al., 2017). In general, almost all statistical models can be used to effectively capture and precisely estimate the additive genetic effect for each marker in a homozygous population. However, they have limited power in evaluating non-additive or non-genetic effects, including dominance, epistatic, and GEI effects when TPs and BPs consist of heterozygous lines and are phenotyped across locations and years (environments). Therefore, optimizing statistical models with consideration of non-additive effects is extremely important to achieve precise marker effect estimation and, thus, high prediction accuracy. By uncovering the pattern of genotype response to different environments, complex trait dissection and performance prediction could be conducted, and a systematic genome-wide performance prediction framework was established (Li et al., 2018). Incorporating GEI effects into statistical models can significantly increase prediction accuracy further when multienvironment trials (METs) are involved (Burgueño et al., 2012, Jarquín et al., 2014, Jarquín et al., 2017, Montesinos-López et al., 2015, Cuevas et al., 2016, Cuevas et al., 2017, Saint Pierre et al., 2016, Millet et al., 2019).

The GS models developed on the basis of a non-linear kernel algorithm, such as reproducing kernel Hilbert space (RKHS), have specific capacity of capturing non-genetic effects and improving the estimated accuracy of marker effects (Gianola et al., 2006, Gianola and de los Campos, 2008, de los Campos et al., 2009, de los Campos et al., 2010). By collecting environmental factors, envirotyping can be used to capture the environment-caused variation and associated GEI (Xu, 2016). Multivariate models have been also proposed, whereby various environments (envirotype parameters) or multiple traits were taken into account simultaneously and multiple datasets were integrated into the models to achieve a better prediction (Guo et al., 2014a, Schulthess et al., 2016, Wang et al., 2017b, Wang et al., 2018, van Eeuwijk et al., 2018). Compared with livestock breeding whereby only several major breeds or varieties are required for well-designed or selected environmental conditions, plant breeding may have to develop many varieties each with the best adaptation to one specific environment or a small area of production region. Therefore, phenotyping under METs and managed environments to tackle GEI can be used in plant breeding to improve GS by constructing training datasets for marker–phenotype association (Cooper et al., 2014b, van Eeuwijk et al., 2018). Using environmental factors to group the target population of environments (TPE), GEI can be characterized for specific production environments or regions, providing a key foundation for creating training datasets for GS (Voss-Fels et al., 2019). Therefore, strategic model design and optimization has become critical to improve prediction accuracy and enhance breeding efficiency in commercial breeding programs.

Non-additive effects include intralocus (dominance) and interlocus (epistasis) non-additivity. With the presence of non-additive effects, breeding populations can have different allele substitution effects at the associated QTL. Both TPs and BPs should be examined to quantify the QTL allele substitution effects for their consistency across populations. With observed predominant consistency, core TPs can be developed to support broad GS application across multiple breeding populations (Cooper et al., 2014b). When no predominant consistency is observed, more TPs or iteratively updated training will be required (Podlich et al., 2004). Large-scale open-source breeding programs ongoing in China, which target hundreds of training and breeding populations (discussed later), can be used to identify the consistent allele substitution effects and the best TP or TP sets to predict specific breeding populations. Prediction accuracy was evaluated using 1831 maize hybrids phenotyped for grain yield and grain moisture across 3 years with genotypes inferred *in silico* based on 207 parental lines genotyped by 500 000 SNPs. Including the dominance effect increased the prediction accuracy for grain production by up to 30%, while the inclusion of interaction effects via multienvironment modeling increased the prediction accuracy overall (Ferrão et al., 2018).

The combined effects of epistasis and GEI can be tackled to enhance genomic prediction for complex traits. As one of the gene-to-phenotype (G2P) models, the crop growth model (CGM) is proposed to capture the combined effects to complement conventional GS (Chenu et al., 2009, Technow et al., 2015). By coordinating a set of biophysical functions, CGM can translate the key environmental variables quantified across different developmental stages into crop growth and development dynamics (Voss-Fels et al., 2019). Combined with whole-genome prediction (CGM-WGP), CGM has been used in three CGM-WGP maize studies (Technow et al., 2015, Cooper et al., 2016, Messina et al., 2018), demonstrating that interactions among intermediate traits in the CGM model interpreted well the non-additive gene actions for yield, and making CGM one of general genomic prediction models. Other biological models that can be used in GS for complex traits include gene network models (Dong et al., 2012) and biochemical and hormone pathway models (Guo et al., 2014b, Marjoram et al., 2014). The former is used to predict the developmental transition while the latter is used to predict the critical levels of development- and adaptation-associated regulators such as metabolites and hormones. CGM-WGP and other alternative G2P modeling methods can be applied to further improve GS by including MET data as additional variables in the prediction model (van Eeuwijk et al., 2018). In addition, the managed-environmental data can be also used to design training datasets to enhance yield stability, as shown in maize (Cooper et al., 2014a).

### Optimizing Prediction Models by Including Other Types of Omics Information

With the development of postgenomic tools and accumulation of omics data, integrating transcriptomic and metabolomic data into the GS models has improved prediction accuracy, because of efficiently capturing minor and non-additive effects especially when hybrid performance was predicted (Westhues et al., 2017, Zenke-Philippi et al., 2017, Schrag et al., 2018; Tables 1 and 2). Multilayered least absolute shrinkage and selection operator (MLLASSO) was developed by including multiple omics data into a single model, enabling the learning of three layers of intermediate variables or genetic features supervised by observed transcriptome and metabolome (Hu et al., 2019). By learning higher-order gene interactions, predictability for rice yield was increased significantly from 0.1588 (genomic prediction alone) to 0.2451 (MLLASSO). Genetically predictable genes, as shown to be predictable accurately with molecular markers, are good predictors for quantitative traits, and are mostly expression QTL genes (cis or trans) with trait-related transcriptional factor families enriched. In maize, genomic, transcriptomic (mRNA and sRNA), and metabolomic data of parent lines (143 Dent and 104 Flint lines) were collected to evaluate the data's ability to predict the agronomic performance of 1567 hybrids. Combining mRNA and genomic data as predictors provided high predictive abilities across both grain yield and grain dry matter content, and combining other predictors improved prediction compared with individual predictors (Schrag et al., 2018). In rice, using 278 hybrids derived from an RIL (recombinant inbred lines) population, the best prediction strategies were determined for yield-related traits by combining omics datasets with different prediction methods. The predictions with integrated genomic and metabolomic data generated better results compared with single-omics predictions (Wang et al., 2019). However, the cost in GS should be seriously considered when the multiple omics data are used in prediction.

## Integrating GS with Modern Breeding Technologies

**Figure 3:**
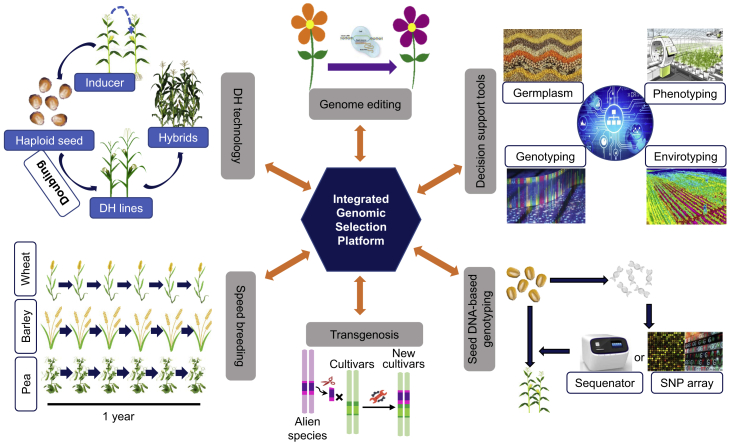
An Integrated Breeding Platform for Genomic Selection. The platform involves various breeding technologies, including doubled haploid (DH) technology, speed breeding, decision support tools, seed DNA-based genotyping, genome editing, and transgenosis.

From the perspective of GS, there are two distinct strategies for enhancing genetic gain in plant breeding. One is to improve prediction accuracy as discussed in the previous section, and the other is to integrate GS with other breeding technologies such as MAS, marker-assisted recurrent selection (MARS), the transgenic approach, genome editing (GE), and doubled haploid (DH) technology, to shorten the breeding cycle time (Table 1; Figures 1 and 3). In addition, GS can be also combined with the speed-breeding method (Watson et al., 2018) to further shorten the breeding cycle time (Hickey et al., 2019). Integrating GS with other functional breeding approaches could create more potential capacity for selecting elite lines that can be used as founders for the next cycle of selection.

### Unlocking and Creating Genetic Variation for Genomic Selection

Genetic variation has been considered as the foundation for breeding selection, which provides genetic resources to accumulate favorable alleles or genes that are linked with targeted traits. There are two approaches that broaden genetic variation for GS: unlocking that which is hidden in genetic resources and creating that which does not exist in our target plants. Hickey et al. (2019) discussed the breeding strategies that integrate DH, speed breeding, GS, and ExpressEdit through CRISPR GE.

Creating more genetic variation may increase both genetic diversity and novel germplasm that can offer more candidates and valuable lines for selection (Xu et al., 2017). In fact, various genetic materials, which include landraces, subspecies, elite lines, ecotypes, and wild relatives, possess abundant genetic variation that should be identified and discovered through traditional genetic analyses or modern techniques based on genomic information and novel statistical models or algorithms, which may involve machine learning or artificial intelligence and depend on big data and powerful servers (Crossa et al., 2017, Grinberg et al., 2018, Montesinos-López et al., 2018; Figure 1 and Table 2). Many approaches can be implemented to identify functional alleles, genes, haplotypes, and networks that broaden the range of genetic variation. Moreover, GE, transgenes, and mutagenesis can be used to transfer or produce novel agronomic traits (Petolino et al., 2016), and subsequently new materials and germplasm can be created for pyramiding favorable alleles, genes, and haplotypes. On the other hand, genome sequencing and pangenome construction can largely unearth favorable genetic variation. Hence, precision and comprehensive reference genomes can ensure that the loci associated with target traits are fine-mapped within true physical locations of each base pair. Pangenomes provide whole-genome coverage and a complete profile of haplotypes and favorable allelic variation, which can be constructed by precise whole-genome sequencing of many and resequencing of large numbers of genotypes (Golicz et al., 2016, Xu et al., 2017). In the context of constructed and existing pangenomes, the first important component for unlocking, creating, and utilizing genetic variation is to discover and clone genes through reverse and forward genetic approaches, and subsequently, the genes and gene regulatory networks should be functionally annotated and constructed to integrate with GS strategy to build up an efficient breeding pipeline.

Transgenic technology and GE are two powerful tools that can be used to improve plant species, the former transferring favorable genes from distant species into crop plants and the latter producing site-specific sequence changes that contribute to improved agronomic traits. Advanced transgene techniques can now stack multiple genes such as those for insect and disease resistance and herbicide tolerance (Sun et al., 2015, Anand et al., 2018, Khabbazi et al., 2018, Lowe et al., 2018, Zhu et al., 2018) into the elite breeding lines developed through other breeding approaches including GS. GE is a type of novel, fast, effective, and precise genetic engineering whereby DNA can be deleted, inserted, modified, or replaced in the target region of the genome (Cho et al., 2013, Bortesi and Fischer, 2015). It has been widely applied in crop plants, and significant examples include those leading to generation of DH lines through maternal haploid induction in maize (Dong et al., 2018), wheat (Liu et al., 2019), and rice (Yao et al., 2018). One of the GE applications in plant breeding is to weed out the deleterious or bad alleles by GE-based targeted mutagenesis, which is not possible in conventional selection due to LD between favorable and deleterious alleles and limited population sizes (Gibson, 2012, Yang et al., 2017, Hirsch and Springer, 2018). Weeding out the bad alleles has been proved in maize by including bad allele information in GS models (Yang et al., 2017) and in cassava by combining the GS with GE to purge the deleterious mutations (Ramu et al., 2017). As proposed by Bernardo (2016), GE can be used to induce targeted recombination breakpoints, by which genetic gain for complex traits in maize could be doubled with predicted marker effects and targeted optimal recombination points throughout the genome. GE can be also utilized for whole-genome editing whereby all the candidate genes with both large and minor effects and, thus, their associated gene networks, could be edited. Integrating GS with the two aforementioned genetic modification approaches will have a huge potential to shorten breeding cycle time. General improvement of complex traits in a crop plant species achieved by GS can be complemented by improving several major-gene controlled traits through genetic modifications. Therefore, improvement of both qualitative and quantitative traits can be achieved simultaneously.

Exotic germplasm that host hidden genetic variation can be harnessed, and relevant genes can be transferred into elite germplasm pools through prebreeding. Such a prebreeding process is more practical in plants than in animals (livestock) (Table 2). Using high-density markers, exotic germplasm can be evaluated for their breeding values and used to reinstate diversity for target traits. Using a large empirical sorghum dataset and a GS-based strategy, gene-bank germplasm was predicted for their performance using a strategically sampled TP (Yu et al., 2016). By stimulated prebreeding with exotic populations, GS has the potential to break large linkage blocks to explore genetic diversity (Cowling et al., 2017). A more recent simulation also demonstrated that a Bayesian optimization algorithm for genomic prediction is potentially useful for prebreeding and would ultimately reduce the accession number required in phenotyping to recover the best genotype (Tanaka and Iwata, 2018).

### Refining Field Management to Improve Heritability Estimation

Heritability has a positive correlation with prediction accuracy of GS (Combs and Bernardo, 2013, Lian et al., 2014, Zhang et al., 2017a). In other words, the higher the heritability estimation an agronomic trait can achieve based on field experimental data in a certain environment, the better the predictive performance will be. In quantitative genetics, heritability refers to the proportion of genetic variance (*V*_g_) in phenotypic variance (*V*_p_), the latter being composed of genetic (*V*_g_) and environmental (*V*_e_) variances. Therefore, refining field management will reduce environmental effects and experimental errors, and thus improve heritability estimation and prediction accuracy (Tables 1 and 2). Environmental variance is largely affected by abiotic factors such as microclimate instability, soil fertility, winds and rainstorms, and biotic factors such as disease pathogens, insects, weeds, and undesired plants and animals around the crop plants. However, various measures can be taken to efficiently manage environments, including consistent crop management, uniform experimental materials, well-selected controls/checks, good border-effect control, reasonable trial design, and field-related techniques such as establishing a wireless sensor network to evaluate and measure climate and soil moisture (Araus and Cairns, 2014, Klukas et al., 2014). In addition, envirotyping should be performed when implementing experiments in managed and field conditions to integrate all information of genotype (G), phenotype (P), and envirotype (E) in a whole-genome strategies as shown in the formula P = G + E (Xu, 2012, Xu, 2016). Furthermore, it is critical to understand GEI by integrating a reasonable CGM with auxiliary information collected with specific agronomic practice and environment management (Technow et al., 2015, Cooper et al., 2016, Xu et al., 2017). Standard and uniform agronomic protocols can greatly contribute to refining field management in order to minimize artificial and environmental errors and improve heritability estimation.

With the development of precision phenotyping, remote sensing, robotics, and artificial intelligence technologies, breeders can perform high-throughput, low-cost, labor-saving precision phenotyping (Araus et al., 2018, Tripodi et al., 2018), which can largely contribute to the enlargement of experimental scale, the reduction of labor requirement, and the removal of human errors in manual measurements. High-throughput precision phenotyping can integrate with other strategies to improve heritability estimation and prediction accuracy (Araus et al., 2018). Therefore, precision agronomic practice and management, refined field trials, and optimized experimental design will significantly improve our capacity to explore minor genetic effects with improved heritability estimation, and subsequently enhance genetic gain.

### Increasing Breeding Scale and Shortening Breeding Cycle Time

The prediction accuracy in GS increases as population size increases, because marker effects can be more efficiently and accurately estimated by statistical models with increased TP size (Crossa et al., 2013, Endelman et al., 2014, Liu et al., 2018). For enlargement of population sizes or experimental scale, the DH technique is a potential choice. Generally there are five approaches to producing haploids in plant breeding (Palmer and Keller, 2005, Xu, 2010). A high-efficiency DH system for haploid induction and chromosome doubling can be developed, as shown in maize using high oil as selection criterion (Melchinger et al., 2013, Dong et al., 2014). The gene related to haploid induction was identified and cloned in maize through fine mapping, targeted segment sequencing, and mutation (Kelliher et al., 2017, Liu et al., 2017), and single nucleus sequencing revealed that sperm DNA fragmentation of haploid inducer around the mitotic stage of pollen development resulted in embryo chromosome elimination (Li et al., 2017). The haploid induction gene discovered in maize has been used for GE to generate DH quickly in maize (Dong et al., 2018), rice (Yao et al., 2018), and wheat (Liu et al., 2019), providing a DH production approach for many crop species. A high-efficiency DH system can provide huge impetus to enlarge experimental scale for heightening selection intensity, as breeding populations can be fixed quickly and more and larger populations can be manipulated simultaneously.

Accelerating breeding programs shortens the breeding cycle time, thus increasing the genetic gain per year. In addition to increasing the breeding scale, the DH breeding procedure has also significantly shortened the breeding cycle time by reducing the time required for reaching homozygosity to only two generations from the eight or more required with conventional breeding approaches (Figure 2). Breeding programs can be also accelerated through speed breeding, an approach that has been proposed and implemented with the management of temperature and supplementary light for culturing four to six generations per year for canola, spring wheat, durum wheat, barley, chickpea, and pea to rapidly obtain stable and heritable candidate lines (Watson et al., 2018; Figure 3). Such speed breeding can be improved or complemented by modifying genes that control and regulate plant growth and development with responses to external environmental conditions and internal stimuli (Hickey et al., 2019, Zhou, 2019).

By GS per se and its combination with DH and other breeding approaches, numerous pure-breeding lines or intermediate breeding materials can be produced or derived. They are too many to be evaluated regularly through field evaluation or test-crossing. A large proportion of candidate individuals can be eliminated or selected before planting or field testing through seed DNA-based MAS (Gao et al., 2008; Figure 3). Such selection can be conducted through regular MAS using candidate genes, functional markers, or favorable haplotypes, or based on individual GEBV estimates using a part of the candidate individuals as TP to develop models for selection of the remainder, as suggested in a previous section. In maize, multinational seed corporations have developed seed-chipping technologies to facilitate seed DNA-based genotyping to preselect DH lines before planting based on both functional markers and GEBVs, significantly reducing the expenditure of subsequent METs.

In hybrid breeding programs, performance prediction of potential crosses can be implemented, and thus a large number of the crosses can be excluded *in silico*, through constructing an appropriate TP and developing valid statistical models that have a capacity to distinguish heterotic groups, estimate general and specific combining ability, and predict hybrid performance, which eventually can offer pertinent recommendations to plant breeding projects (Figure 2). As an extension to GS, optimal haploid value (OHV) selection, was proposed to predict the best DH that could be produced from a segregating plant (Daetwyler et al., 2015), which is implemented by focusing on haplotype selection and optimizing the breeding program toward its end-product—an elite fixed line. Rigorous testing using computer simulation revealed up to 0.6 standard deviations more genetic gain than GS. On the other hand, OHV selection preserved a substantially higher level of genetic diversity in the population than GS for long-term genetic gain. By introducing *in vitro* nurseries into rapid generation advancement, genotyping can be done on gametes or new cell lines (La Fuente et al., 2013). This idea has been tested with an extremely fast-turnaround GS to shorten breeding cycle time significantly, as shown in selection of cattle embryos (Shojaei Saadi et al., 2014) and as expected for *in vitro* selection of desirable DH lines.

Integrated breeding platforms would contribute to improved breeding efficiency and enlarged experimental scale to heighten selection intensity that will eventually enhance genetic gain. When combined with MAS, for example, the DH approach results in increased genetic gain by facilitating multiple trait and gene stacking, increased efficiency and probability of successful variety development, and reduction in the time to market. Therefore, multidisciplinary collaboration can be explored to construct a well-managed, highly efficient, and maneuverable plant breeding system to provide sufficient information for breeding elite lines with the purpose of acquiring higher genetic gain (Xu et al., 2017; Figure 3).

## Establishing an Open-Source Breeding Network for Genomic Selection

### Why We Need an Open-Source Breeding Network

Increasing genetic gain in breeding programs has been driven by increased resource inputs, and the cost-benefit balance determines how modern breeding technology can be eventually employed. Overall breeding cost includes establishing, maintaining, and utilizing various breeding platforms, such as those required in genotyping, phenotyping, envirotyping, information management, and decision support (Figures 1 and 3; Xu et al., 2017). As reviewed by Spindel and McCouch (2016), many studies have revealed that the more correlated are the phenotypic and environmental data used to train GS models, the better are the prediction accuracies and the more useful the breeding outcomes that can be achieved, which was also confirmed in wheat GS breeding (Battenfield et al., 2016). In multinational breeding companies, GS has been implemented and supported with one set of well-equipped and centralized platforms, achieving significant cost efficiency due to large-scale and standardized protocols and applications. In developing countries, however, the public sector and small- and medium-sized companies, each running independently, greatly suffer from limited funding and resources that can be allocated to less-equipped facilities and poorly supported service. To make GS breeding programs practical in this case, therefore, an open-source breeding network should be established for sharing various resources including facilities, platforms, and breeding-related data across GS breeding programs (Table 2).

Collected data, which should be freely available within an open-source breeding initiative, include genotypes, phenotypes, and envirotypes that are generated for additional genotypes of the same population, more populations with related parents, the same populations that are tested in additional environments (seasons, years, or locations), or some combinations thereof. Open-source breeding provides numerous opportunities of using existing and accumulated genotypic and phenotypic data created worldwide in the public sector (e.g., Juliana et al., 2019) or GS consortia to identify or develop the best-fit model and TP for each BP. To make the data sharable and updatable, common standards, vocabularies, and data structure should be adopted and training data should be collected from a wide range of breeding programs (Fiorani and Schurr, 2013, Krajewski et al., 2015, Spindel and McCouch, 2016).

### Sharable and Flexible GS Breeding Platforms

Genotyping cost usually accounts for a large proportion of overall breeding cost, determining how GS could be implemented. By sharing genotyping platforms and running numerous samples in genotyping, multinational seed companies have achieved a significant cost advantage (up to 50%–70% savings) compared with individual breeding programs in developing countries. Establishing sharable genotyping platforms becomes one of the best options to reduce genotyping-related costs. Such sharable GS breeding platforms should be established for all GS-related components, including phenotyping, envirotyping, information management, and decision support tools (Table 2). The platforms that can be shared should be standardized with high capacity and multiple functions or purposes, suitable for different plant or even animal species. Compared with other components, phenotyping could be most difficult for standardization and thus would be less sharable across different plant species. In addition to shareability, GS breeding platforms should be also flexible enough to make one platform functional for multiple purposes. Taking a genotyping platform as an example, it may change with the development of sequencing technology, and the final genotyping platform would be whole-genome sequencing with the reads long enough to cover long-range repeat regions so that little bioinformatics effort would be required for data processing and analysis. Significant technical advances are required for highly automatic sequencing and assembly of the whole genome at very low cost. To this end, selective and targeted sequencing is more desirable.

Reduced-representation GBS (Scheben et al., 2017) or skim-based GBS (Bayer et al., 2015) needs significant bioinformatics support and heavy imputation, as well as overcoming the difficulties in data sharing and comparing across users and labs. Targeted sequencing integrated with highly multiplexed PCR has generated a highly cost-effective genotyping platform, called GBTS, which consists of two marker models: multiplex PCR (GenoPlexs) for several to 5K markers and in-solution capture (GenoBaits) for 1K–45K markers. The latter has been used for development of a 20K marker panel, from which three other marker panels (10K, 5K, and 1K) could be generated by sequencing at the average sequencing depths of 20×, 7.5×, and 2.5×, respectively (Guo et al., 2019a). Now in-solution capture has been optimized and upgraded to genotype 40K mSNPs, each mSNP containing a cluster of multiple (4–8) SNPs with a total of 260K SNPs. Using the same set of 40K mSNPs, various numbers of SNPs can be generated by sequencing at different depths (Z. Guo, J. Zhang and Y.X., unpublished data). Therefore, the GBTS system provides a very flexible and also affordable genotyping platform for marker-assisted breeding including GS. Compared with the genotyping platform the phenotyping platform is less flexible, while other platforms such as envirotyping, informatics, and decision support are already flexible enough.

### Open-Source Breeding Networks

**Figure 4:**
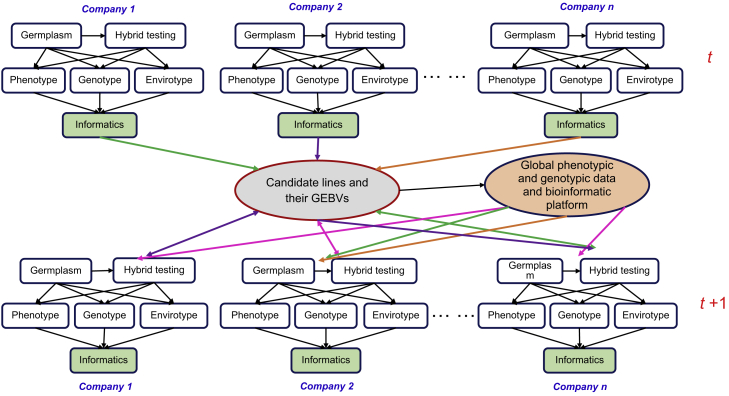
Open-Source Breeding and Genomic Selection Networks Provide Services to Developing Countries and Small- and Medium-Sized Breeding Companies. All breeding-related information, including estimated marker effects and genomic estimated breeding values (GEBVs), even breeding materials, can be shared during the breeding process and after each breeding cycle (*t* and *t* + 1), functioning in the same way as in a multinational seed incorporation where each breeding team works as a small- and medium-sized company (1, 2, …, *n*).

The scale of breeding programs in developing countries and small- and medium-sized breeding companies is markedly smaller than that in developed countries and multinational incorporations, so that molecular breeding platforms such as those for genotyping, phenotyping, and envirotyping, once established, will be left unused for most of the time unless they are shared across companies, institutions, or countries. Therefore, an open-source GS breeding network, combined with shared molecular breeding platforms, needs to be built up, which has the capacity of providing national agricultural research organizations and small- and medium-sized breeding companies with advanced and comprehensive breeding technologies, including a high-throughput genotyping platform, improved phenotyping capacity, integrated germplasm resource management, and well-established modeling and prediction approaches that are now only available and functional in multinational breeding companies (Figure 4). The users linked by the network will keep all the platforms running full time, resulting in significantly reduced unit costs. Such an open-source breeding network can be shared across animal and plant breeding programs, the two fields that have diverged but can now be unified through GS (Hickey et al., 2017).

An open-source breeding network can be viewed as part of a high-efficiency breeding pipeline, and has at least four general advantages: (1) the ability to share phenotypic information between network members; (2) providing synthetic pipeline services and genotypic data between members with partnership; (3) low-cost genotyping platforms; (4) the capacity to obtain haplotype effects among environments and traits that cannot be analyzed individually (Xu et al., 2017). With the development of modern technologies in plant breeding, the cost of genotyping has been dramatically reduced in comparison with phenotyping expenditure (Guo et al., 2019a), and it has made marker-based individual evaluation come true, which contributes to the generalization and application of GS strategy in commercial breeding programs. As an example from the agriculture industry in a developing country, an excellent global open-source breeding program, the Consortium of International Agricultural Research Centers (CGIAR), is engaged in researching a quantity of animal and plant species, and can synergistically integrate its global resources and expertise with GS within its network in terms of prebreeding, conventional breeding, and molecular breeding. This project can integrate small breeding programs in small- and medium-sized breeding companies to efficiently and rapidly utilize their latest progress and to share their resources and information for the achievement of greater genetic gain (Hickey et al., 2017; Figure 4).

As the first large-scale public sector effort, the Genomic Open-Source Breeding informatics initiative (GOBii; http://gobiiproject.org/) has been established for systematically applying high-density genotypic information to plant breeding. Open-source genomic data management and analysis tools have enabled breeders to integrate their breeding programs with GS and other MAS Strategies. As a support to the open-source breeding network, CGIAR established the Excellence in Breeding Platform (http://excellenceinbreeding.org), which consists of six components: breeding program excellence; trait discovery; breeding tools and services; genotyping and sequencing; phenotyping; and bioinformatics and data management. Another component that should have been included is envirotyping for collecting various sources of environmental data and their use in plant breeding (Figures 3 and 4). In China, where crop breeding has been done independently by individual institutions, universities, and small- and medium-sized breeding companies, a great effort has been made to establish national molecular breeding networks or initiatives, supported by national genotyping facilities or service providers. In maize, GS for 100 biparental populations has been initiated and will be completed in 3 years, whereby TPs, developed models, and marker effects can be shared across China and the best training model can be developed for each specific breeding population. At CIMMYT a reference wheat genotype–phenotype map has been built, and 44 624 wheat lines have been fingerprinted using GBS, with over 7.6 million data points generated in genotyping and a large number of marker-trait associations identified (Juliana et al., 2019), providing a valuable resource for open-source breeding for the worldwide wheat community. In other cases, genetic and breeding materials can be also shared along with developed genetic models and estimated marker effects, by fingerprinting the shared germplasm before release so that breeders' contribution to newly developed lines can be estimated by fingerprinted parental or donor lines. As an early example in plant breeding, the Open Source Seed Initiative (OSSI) (https://osseeds.org/), recruits a group of excellent plant breeders, industrious farmers, seed enterprises, nonprofit organizations, and policymakers for the purpose of maintaining and promoting an available open-source gene bank that can share the plant genetic resources and germplasm among participants around the world. OSSI can provide all kinds of and accessible opportunities for breeders to release newly developed lines or breeding populations compiled by the OSSI pledge, for which the cultivars or varieties should be unique and have been developed based on different heterotic groups (Luby et al., 2015).

Molecular breeding networks, supported by open-source breeding, have been contributing to large-scale GS practice. Taking CIMMYT as an example, a total of 7956 DH or F5:6 lines were used in maize GS, among which 1926 lines phenotyped in 3 years were used as TP, and 5030 lines never phenotyped were used as a breeding/prediction population. Finally, 587 of the 5030 lines (11.7%) were selected based on GEBVs and recommended to breeders for further testing and validation. Selection intensity in GS was doubled compared with that used in phenotypic selection, around 20% in Stage-1 testing. The total genotyping cost for running this study is US$25 000 with a subsidy of $3.5 per sample from Bill and Melinda Gates Foundation, while the full genotyping cost without any subsidy is around $40 000, which is equivalent to the cost of phenotyping 1000 lines in three locations each with two replications, according to the current cost at CIMMYT, $7.00 per plot. Therefore, the tested population size in this GS study increased eightfold compared with the phenotypic selection (X. Zhang and M.S.O., unpublished). The coordination and communication to enable delivery against tight deadlines is critical in open-source breeding programs. This testing of GS at scale at CIMMYT has the potential to affect many more breeding programs through the Excellence in Breeding Platform. This was a sizeable, real-time GS application in public-sector breeding programs serving low- and middle-income countries, and breeders will be interested in what we learn from this process. Hence, a breeding program of integrating GS with other available approaches and tools should be established to assist developing countries, public sectors, and small- and medium-sized enterprises to augment efficiency and the level of breeding, and thus enhance the genetic gain in farmers' fields.

## Perspectives

The efficient and precise GS pipeline should be constructed for achieving and obtaining greater genetic gain and for improving the production of staple crops to meet the human demand from an increasing global population. In the era of molecular breeding, GS as a prominent and promising strategy will become an increasingly widespread application in plant breeding, as in livestock, with the evolution of key GS components and associated platforms. With the development of cost-effective genotyping platforms and high-efficiency breeding strategies, GS-assisted breeding will spread from livestock to plants and from case applications in few crops for some traits to wide applications in all major crop plants for all important traits, from individual regions to countries worldwide, and from isolated private sectors to associated partners through open-source breeding networks. The collaboration between GS and other technologies or transdisciplinary approaches is extremely important for developing a high-efficiency breeding pipeline in terms of rapidly pyramiding major genes identified by QTL mapping or GWAS into targeted lines. Besides, a set of closely linked genes within the chromosome can be inherited together as a haplotype and integrally transmitted from parental or ancestral lines to offspring. The concept of haplotype can be extended from the level of a single region within a chromosome to the whole genome to cover many functional markers. To enhance genetic gain by GS, genomics-assisted tools should be utilized to create a chimera that contains optimized combinations and haplotypes of two or more elite parental lines. GS-assisted breeding programs can be implemented with the reference of haplotypic effects, from which the accuracy of GS prediction can be improved and the associated breeding pipeline can be optimized to stack favorable genes into one elite line to create excellent varieties or hybrids. By incorporating more and more known genes and their favorable haplotypes, the blocks with pyramided favorable genes and networks for the target trait and trait combinations can be built up. Comparative analysis of GS results with long-term phenotypic selection for protein and oil contents that have been running for over 100 generations (Goldman et al., 1993, Laurie et al., 2004, Li et al., 2013) and with selection of heterotic groups for several decades (Duvick et al., 2004, Lee and Tracy, 2009) will help us to understand the responses and advantages of GS. All these efforts will facilitate the development of new breeding strategies and methodologies to enhance genetic gain. In commercial breeding companies, integrated plant breeding platforms will be conducive to raising efficiency and balancing cost-benefit for further enhancement of genetic gain. However, partnership or consortia, such as open-source breeding networks, will have great potential and a bright future among small enterprises that can make the best use of their respective advantageous resources to integrate with a GS strategy to accelerate the breeding process through sharing breeding platforms and information. Low-cost and high-throughput genotyping platforms that become increasingly available will help remove one of the key constraints that stop GS and other MAS methods from being using on a large scale. The next challenge will be to make GS a routine practice by implementing various steps into an efficient analytical pipeline. Multinational breeding companies have accumulated a large amount of historical data during their long-term breeding programs, and their data analysts can adequately use historical data as a way of amplifying the experimental scale to implement GS for increased prediction accuracy (Xu, 2018, Hao et al., 2019). A synthetic breeding system, which may mainly focus on GS, should be established in plant breeding programs with the development of GS theory and associated breeding platforms. Several large-scale GS breeding programs ongoing in China may provide convincing examples to support open-source breeding programs that can significantly enhance genetic gain.

## Funding

The research involved in this report was supported by the 10.13039/501100012166National Key Research and Development Program of China (2016YFD0101803), the 10.13039/501100012166National Key Basic Research Program of China (2014 CB138206), the 10.13039/501100012421Agricultural Science and Technology Innovation Program of 10.13039/501100005196CAAS, and Fundamental Research Funds for Central Non-Profit of Institute of Crop Sciences, CAAS (1610092016124). Research activities of CIMMYT staff have been supported by the 10.13039/100000865Bill and Melinda Gates Foundation and the CGIAR Research Program MAIZE.

## Supplemental Information

Document S1. Supplemental Table 1 and Supplemental References
