# Supplementary material for: Enhancing Genetic Gain through Genomic Selection: From Livestock to Plants
Source: Plant Commun. 2019 Oct 16;1(1):100005. doi: 10.1016/j.xplc.2019.100005 (PMC7747995; doi:10.1016/j.xplc.2019.100005)
Supplement: Document S1. Supplemental Table 1 and Supplemental References [file mmc1.docx]

| **Crop and population type** | **Population size (marker number)** | **Traits** | | **Models** | **Accuracy** | **Ref** |
| --- | --- | --- | --- | --- | --- | --- |
| **Wheat** |  | |  |  |  |  |
| NP | 483(34,095) | Plant height, powdery mildew, grain yield | | RR-BLUP | 0.42-0.72 | 1 |
| NP | 1604(17,372) | Grain yield | | GBLUP | 0.33-0.64 | 2 |
| F_2:4_ | 2992(24,498) | Yield-related traits | | GBLUP | 0.025-0.30 | 3 |
| NP | 438-585(5831-8398) | Heading date, disease resistance, plant height | | RR-BLUP | 0.60-0.91 | 4 |
| NP | 269(39,856) | Grain yield and nutrients | | GBLUP, RKHS, RR-BLUP, RF, EN, LASSO | -0.22-0.52 | 5 |
| NP | 264 and 268(33,762) | Grain yield, plant height, leaf rust | | BL, RKHS | 0.52-0.79 | 6 |
| **Maize** |  |  | |  |  |  |
| NP | 966(2911) | Yield, test weight | | RR-BLUP | 0.12-0.36 | 7 |
| NP | 1970(37,479) | Grain yield, grain dry matter content | | GBLUP | 0.10-0.80 | 8 |
| NP | 906(26,637-34,571) | Grain yield, plant height, stay green | | GBLUP, CNV | 0.31-0.71 | 9 |
| NP | 240(29,619) | Grain yield, ear girth, kernel weight, ear length, kernel number | | RR-BLUP, BL, RF, BA, BB, EN, RKHS | 0.28-0.92 | 10 |
| **Rice** |  |  | |  |  |  |
| Immor-  talized F_2_ | 278(1619) | Yield, tiller, 1000 grain weight, grain# per panicle | | LASSO, BayesB, SVM, PLS | 0.05-0.70 | 11 |
| RIL | 210(270,820) | Yield-related traits | | LASSO, MLLASSO | 0.12-0.76 | 12 |
| NP | 575-3023(116,482-2,054,293) | Yield-related traits | | GBLUP, BayesB, LASSO, SVM | 0.38-0.88 | 13 |
| NP | 575-3023 (2,395,866- 3,299,150) | Grain yield per plant, grain weight, grain number, etc. | | GBLUP | 0.39-0.88 | 14 |
| NP | 363(108,005) | Plant height, flowering time, maturity date, lodging score, grain yield | | RR-BLUP, BL, RKHS, RF, MLR | -0.34-0.70 | 15 |
| **Sorghum** |  |  | |  |  |  |
| NP | 2645(4781) | Grain yield, stay green, plant height, flowering time | | GBLUP, ABLUP | 0.16-0.58 | 16 |
| **Soybean** |  |  | |  |  |  |
| NP | 309(31,045) | Seed weight | | RR-BLUP | 0.62-0.85 | 17 |
| **Pea** |  |  | |  |  |  |
| RIL | 105×3(479-514) | Flowering time and grain yield | | RR-BLUP, BL, SVM | 0.25-0.72 | 18 |
| **Barley** |  |  | |  |  |  |
| F_4:5_ | 647(1,536) | Deoxynivalenol accumulation, *Fusarium head blight*, yield | | RR-BLUP | 0.03-0.99 | 19 |
| **Tomato** |  |  | |  |  |  |
| NP | 163(5,995) | 35 metabolic traits | | RR-BLUP | 0.05-0.81 | 20 |

Supplemental Table 1. Significant reports on genomic selection in crop plants. NP: natural population; DH: doubled haploid; RIL: recombinant inbred line; GBS: genotyping-by-sequencing; SNP: single nucleotide polymorphism; GBLUP: genomic best linear unbiased prediction; RKHS: reproducing kernels Hilbert spaces regression; RR-BLUP: ridge regression best linear unbiased prediction; LASSO: least absolute shrinkage and selection operator; BL: Bayesian LASSO; RF: random forest; EN: elastic net; BA: BayesA; BB: BayesB; PBLUP: pedigree-based best linear unbiased prediction; MLR: multiple linear regression; SVM: support vector machine; PLS: partial least squares.

References

1. Sarinelli, J.M., Murphy, J.P., Tyagi, P., Holland, J.B., Johnson, J.W., Mergoum, M., Mason, R.E., Babar, A., Harrison, S., Sutton, R., et al. (2019). Training population selection and use of fixed effects to optimize genomic predictions in a historical USA winter wheat panel. Theor. Appl. Genet. 132: 1247-1261.
2. Guo, T., Yu, X., Li, X., Zhang, H., Zhu, C., Flint-Garcia, S., McMullen, M.D., Holland, J.B., Szalma, S.J., Wisser, R.J., et al. (2019). Optimal designs for genomic selection in hybrid crops. Mol. Plant 12: 390–401.
3. Edwards, S.M., Buntjer, J.B., Jackson, R., Bentley, A.R., Lage, J., Byrne, E., Burt, C., Jack, P., Berry, S., Flatman, E., et al. (2019). The effects of training population design on genomic prediction accuracy in wheat. Theor. Appl. Genet. 132: 1943-1952.
4. Herter, C.P., Ebmeyer, E., Kollers, S., Korzun, V., Würschum, T., Miedaner, T. (2019). Accuracy of within- and among-family genomic prediction for Fusarium head blight and Septoria tritici blotch in winter wheat. Theor. Appl. Genet. 132: 1121–1135.
5. Manickavelu, A., Hattori, T., Yamaoka, S., Yoshimura, K., Kondou, Y., Onogi, A., Matsui, M., Iwata, H., Ban, T. (2017). Genetic nature of elemental contents in wheat grains and its genomic prediction: toward the effective use of wheat landraces from Afghanistan. PLoS ONE 12: e0169416.
6. Habyarimana, E. (2016). Genomic prediction for yield improvement and safeguarding of genetic diversity in CIMMYT spring wheat (*Triticum aestivum* L.). Aust. J. Crop Sci. 10: 127.
7. Brandariz, S.P., Bernardo, R. (2019). Small ad hoc versus large general training populations for genomewide selection in maize biparental crosses. Theor. Appl. Genet. 132: 347–353.
8. Schrag, T.A., Schipprack, W., Melchinger, A.E. (2019). Across-years prediction of hybrid performance in maize using genomics. Theor. Appl. Genet. 132: 933–946.
9. Lyra, D.H., Galli, G., Alves, F.C., Granato, Í.S.C., Vidotti, M.S., Bandeira e Sousa, M., Morosini, J.S., Crossa, J., Fritsche-Neto, R. (2019). Modeling copy number variation in the genomic prediction of maize hybrids. Theor. Appl. Genet. 132: 273–288.
10. Shikha, M., Kanika, A., Rao, A.R., Mallikarjuna, M.G., Gupta, H.S., Nepolean, T. (2017). Genomic selection for drought tolerance using genome-wide SNPs in maize. Front. Plant Sci. 8, 550.
11. Wang, S., Wei, J., Li, R., Qu, H., Chater, J.M., Ma, R., Li, Y., Xie, W., Jia, Z. (2019). Identification of optimal prediction models using multi-omic data for selecting hybrid rice. Heredity 123: 395-406.
12. Hu, X., Xie, W., Wu, C., Xu, S. (2019). A directed learning strategy integrating multiple omic data improves genomic prediction. Plant Biotechnol. J. 17: 2011-2020.
13. Xu, Y., Wang, X., Ding, X., Zheng, X., Yang, Z., Xu, C., Hu, Z. (2018). Genomic selection of agronomic traits in hybrid rice using an NCII population. Rice 11: 32.
14. Wang, X., Li, L., Yang, Z., Zheng, X., Yu, S., Xu, C., Hu, Z. (2017). Predicting rice hybrid performance using univariate and multivariate GBLUP models based on North Carolina mating design II. Heredity 118: 302–310.
15. Spindel, J.E., Begum, H., Akdemir, D., Collard, B., Redoña, E., Jannink, J.-L., McCouch, S. (2016). Genome-wide prediction models that incorporate *de novo* GWAS are a powerful new tool for tropical rice improvement. Heredity 116: 395-408.
16. Velazco, J.G., Malosetti, M., Hunt, C.H., Mace, E.S., Jordan, D.R., van Eeuwijk, F.A. (2019). Combining pedigree and genomic information to improve prediction quality: an example in sorghum. Theor. Appl. Genet. 132: 2055-2067.
17. Zhang, J., Song, Q., Cregan, P.B., Jiang, G.-L. (2016). Genome-wide association study, genomic prediction and marker-assisted selection for seed weight in soybean (*Glycine max*). Theor. Appl. Genet. 129: 117–130.
18. Annicchiarico, P., Nazzicari, N., Pecetti, L., Romani, M., Ferrari, B., Wei, Y., Brummer, E.C. (2017). GBS-based genomic selection for pea grain yield under severe terminal drought. Plant Genome 10: 1–13.
19. Sallam, A.H., Endelman, J.B., Jannink, J.-L., Smith, K.P. (2015). Assessing genomic selection prediction accuracy in a dynamic barley breeding population. Plant Genome 8: 1–15.
20. Duangjit, J., Causse, M., Sauvage, C. (2016). Efficiency of genomic selection for tomato fruit quality. Mol. Breeding 36: 29.
